# Taste and Smell Disorders: A Critical Look at Olfactory and Gustatory Dysfunction

**DOI:** 10.3390/life14030301

**Published:** 2024-02-26

**Authors:** Antonino Maniaci, Jérome R. Lechien, Luigi Angelo Vaira, Luigi La Via

**Affiliations:** 1 Faculty of Medicine and Surgery, University of Enna “Kore”, 94100 Enna, Italy; tnmaniaci29@gmail.com; 2 Department of Anatomy and Experimental Oncology, Mons School of Medicine, UMONS Research Institute for Health Sciences and Technology, University of Mons (UMons), 7000 Mons, Belgium; jerome.lechien@umons.ac.be; 3 Department of Otolaryngology-Head Neck Surgery, Elsan Polyclinic of Poitiers, 86000 Poitiers, France; 4 Maxillofacial Surgery Operative Unit, Department of Medicine, Surgery and Pharmacy, University of Sassari, 07100 Sassari, Italy; lavaira@uniss.it; 5 Biomedical Science Department, PhD School of Biomedical Science, University of Sassari, 07100 Sassari, Italy; 6 Department of Anesthesia and Intensive Care, University Hospital Policlinico “G. Rodolico-San Marco”, 95123 Catania, Italy

## 1. Introduction

**Figure 1:**
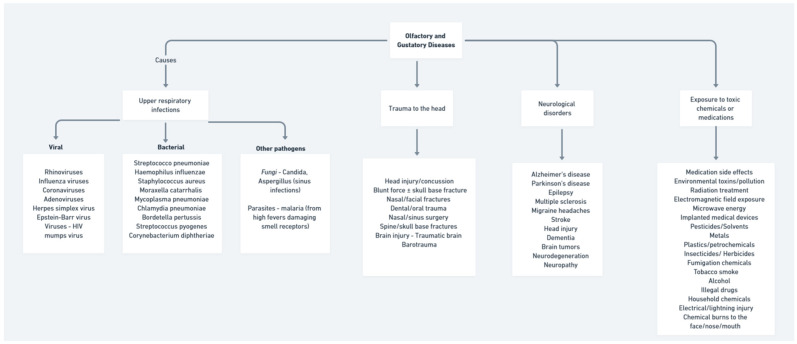
Flow diagram—principal causes of olfactory and gustatory disease.

In an era where modern medicine has made remarkable advances in managing diseases in the head and neck region, we present this Special Issue to provide a spotlight on the new research advances on olfactory and gustatory disorders. In the United States, there are currently 2.7 million adults with an olfactory dysfunction and 1.1 million adults with a gustatory dysfunction. These dysfunctions are also known to have a higher prevalence in older adults, with more than half of those between the ages of 65 and 80 years and more than 75% of those over 80 years old having a demonstrable decline in their sense of smell [1,2] These conditions, which affect the vital human senses of smell and taste, have unfortunately been overlooked and overshadowed amidst the focus on more widely-known diseases and treatment breakthroughs [3,4,5,6]. However, disorders of smell and taste deserve dedicated attention and research, as they can severely impact patients’ quality of life, safety, nutrition, and emotional well-being [7]. The intricate biology and physiology behind our abilities to smell and taste make these senses susceptible to disruptions from infections, injuries, neurodegenerative conditions, and environmental exposures (Figure 1) [8,9,10]. Impairments in smell and taste are warning signs of underlying illness and can serve as harbingers of further disease progression if not addressed. At the same time, loss of smell or taste, even temporarily, can detach people from the joy of flavors, aromas, and fond memories associated with these senses. Yet these substantial effects on patients’ lives are often underestimated or dismissed as being less concerning than other medical issues that determine their cause, especially after the recent COVID-19 pandemic [11,12,13,14,15,16].

## 2. The Critical Role of Smell and Taste

The human olfactory and gustatory systems are remarkably intricate and delicately balanced. These chemical senses are indispensable in allowing us to enjoy life’s pleasures through flavors and scents. Our abilities to smell and taste also serve critical functions in our health, safety, nutrition, and social interactions [17]. The olfactory system detects thousands of odors via receptors in the nasal cavity, transmitting signals about food, environments, and potential threats (e.g., gas, smoke) to the brain. The gustatory system recognizes five primary taste modalities—sweet, salty, sour, bitter, and umami—through taste buds on the tongue and palate. Together, smell and taste provide detailed sensory information about the world around us. Disorders in these systems can therefore be seriously detrimental. Impairments in smell or taste can be early harbingers of neurodegenerative diseases like Alzheimer’s and Parkinson’s, as these senses are closely linked to central nervous system function [18]. Smell and taste deficits may also be manifestations of systemic illnesses such as diabetes, hypertension, autoimmune disorders, or vitamin deficiencies. Environmental triggers like air pollution, smoking, or toxic exposures can also damage smell and taste. Yet, despite these diverse causes, the functional impact of smell and taste disorders is often deeply underestimated or overlooked [19]. These conditions can lead to nutritional deficits, unsafe food choices, an inability to detect environmental hazards, social isolation, and emotional distress. This considerable effect on quality of life underscores why smell and taste merit greater clinical attention and research. 

## 3. Emerging Research and Advances in Diagnosis and Treatment

In this Special Issue, we shed light on the complexities of olfactory and gustatory disorders by bringing forth the latest research, clinical insights, and technological advances in diagnosing, treating, and managing these conditions. A particular focus is the recent surge in chemosensory dysfunctions associated with viral infections, including the smell and taste loss observed globally with COVID-19 [20]. This pandemic brought unprecedented attention to these sensory deficits, highlighting the need for more research and improved clinical care. However, even before COVID-19, progress was being made. Novel diagnostic methods like psychophysical olfactory testing, gustatory evoked potentials, and objective biomarkers of nerve damage are enhancing our ability to identify and characterize smell and taste deficits [21]. Meanwhile, emerging treatments ranging from corticosteroids and olfactory training to platelet-rich plasma and neuromodulation are providing new options for sensory rehabilitation [22,23,24,25].

## 4. Looking to the Future

In curating this Special Issue, we aim to provide an inclusive platform for productive dialogue among scientists, clinicians, and patients affected by smell and taste disorders. Bringing together these diverse perspectives will enable shared learning and spark innovative ideas to advance the field. We envision a future where sensory health is given its due attention within medicine and science, leading to improved diagnostic rates, treatment options, and patient outcomes. We should therefore consider this Special Issue to be a step towards the future evolution of this clinical picture. We are sure that readers will find this edition and future updates useful.
